# Understanding the Heterogeneity of Human Pericyte Subsets in Blood–Brain Barrier Homeostasis and Neurological Diseases

**DOI:** 10.3390/cells10040890

**Published:** 2021-04-14

**Authors:** Diana G. Bohannon, Danielle Long, Woong-Ki Kim

**Affiliations:** 1 Department of Microbiology and Molecular Cell Biology, Eastern Virginia Medical School, Norfolk, VA 23507, USA; BohannDG@evms.edu (D.G.B.); LongD@evms.edu (D.L.); 2 Center for Integrative Neuroscience and Inflammatory Diseases, Eastern Virginia Medical School, Norfolk, VA 23507, USA

**Keywords:** Alzheimer’s disease, blood–brain barrier, endothelial cell, laminin, multiple sclerosis, pericyte, perivascular macrophage, sonic hedgehog, vascular smooth muscle cell

## Abstract

Pericytes are increasingly recognized as being important in the control of blood–brain barrier permeability and vascular flow. Research on this important cell type has been hindered by widespread confusion regarding the phenotypic identity and nomenclature of pericytes and other perivascular cell types. In addition, pericyte heterogeneity and mouse–human species differences have contributed to confusion. Herein we summarize our present knowledge on the identification of pericytes and pericyte subsets in humans, primarily focusing on recent findings in humans and nonhuman primates. Precise identification and definition of pericytes and pericyte subsets in humans may help us to better understand pericyte biology and develop new therapeutic approaches specifically targeting disease-associated pericyte subsets.

## 1. Introduction

Pericyte biology is a growing field which focuses on the role of pericytes (PCs) in vascular homeostasis and disease. While PCs can be found surrounding microvasculature throughout the body, they are of particular importance to the blood–brain barrier (BBB) where they surround endothelial cells (ECs) and, in conjunction with astrocytes, help to establish a selectively permeable cellular system. A majority of research on PCs and the BBB uses mice and mouse models of diseases, and relatively little neurovascular research is conducted in humans. Mice may be a more accessible model; however, studies suggest that differences within the vascular anatomy of mice when compared to humans, or even other strains of mice, may make it difficult to make side-by-side comparisons [1,2]. It is becoming increasingly important to consider new and meaningful ways to investigate the role of vascular elements, such as microvascular PCs, in human tissue. Some of the roles that PCs play in BBB homeostasis and brain pathology are already known and can be further defined through closer investigations.

Under physiological conditions, PCs produce extracellular matrix and other proteins which contribute to the formation of basement membranes and regulate BBB homeostasis [3,4]. Additionally, PCs play an important role in promoting production of tight junction proteins (TJPs), which are essential for creating the tight seams found between ECs of the BBB [5,6]. While PCs affect endothelial tight junction formation via several pathways including transforming growth factor-β1/SMAD signaling [7], it was recently shown that the Sonic Hedgehog (Shh) signaling pathway in PCs may mediate the effect of PCs on TJP production by ECs [8].

The Shh pathway supports the selective permeability of the BBB by promoting the upregulation of TJP production by activating the transcription factor GLI1 [9]. Shh is secreted in a soluble form from astrocytic endfeet into the BBB, where it then binds to patched-1 receptors on the EC surface, releasing smoothened to activate GLI1-induced TJP transcription, but recently other contributors, produced in PCs, to this pathway have been discovered [10,11,12]. Developing a deeper understanding of how PCs work to regulate BBB homeostasis could ultimately lead to therapeutic advances in maintaining the ideal homeostatic conditions of the neurovasculature, thus preventing or reducing the pathological effects of BBB breakdown.

In addition to their role in maintaining BBB homeostasis, PCs have been implicated in pathological processes leading to many neurological disorders. The normal production of pericytic laminin-211 is involved in oligodendrocyte progenitor cell maturation during remyelination, and a lack of PCs or an inability for PCs to produce laminin-211 can result in myelin defects [13,14]. Based on these data, PCs are currently being considered as a new therapeutic target for treatment of demyelinating diseases such as multiple sclerosis (MS). Similarly, like ECs, PCs have been shown to express low density lipoprotein receptor-related protein 1 (LRP1), which, in conjunction with apolipoprotein E (apoE), can transport amyloid beta (Aβ) across the BBB as an export mechanism to remove it from the brain parenchyma [15]. This could indicate a role for PCs in Alzheimer’s disease. While PCs are being found in association with these and other diseases and disorders of the brain, it is still unclear whether specific PC subsets may play different roles in vivo, or whether PCs can change their phenotype or function under pathological conditions. To further examine the role of PCs in BBB homeostasis and disease, we must first investigate methods to differentiate PCs from other perivascular cell types in the human brain.

## 2. Distinguishing PCs from Other Perivascular Cell Types in the Human Brain

One of the most challenging aspects of pericyte biology has been correctly identifying PCs and differentiating them from other cells within the neurovascular niche. While electron microscopy (EM) can often be used to successfully identify PCs, large-scale light microscopy identification of PCs has proven much more difficult. Misuse of cell makers over the decades has convoluted literature in the field, making it more difficult to correctly attribute roles to PCs or other cells within the perivascular niche, but achieving this distinction is key to understanding their unique contributions to health and disease.

### 2.1. Distinguishing PCs from Perivascular Macrophages

Historically, there has been some confusion differentiating between PCs and macrophages in the perivascular regions of the brain. In early studies of the brain, macrophages in the perivascular location, which are now called perivascular macrophages (PVMs), were mistaken as granular PCs due to their EM appearance surrounding the cerebral capillaries, only to be rectified later when it was determined that these PVMs and PCs inhabit different perivascular regions [16,17]. Additional confusion arose in the 1980s when an entire population of MHC class II+ cells, which were not PCs, were identified within the perivascular or Virchow-Robin spaces, and were then collectively called perivascular cells or Mato’s fluorescent granular perithelial cells [18,19]. Other studies sought markers to differentiate between PCs and PVMs, but many were fraught with misconceptions and technical limitations. PCs are often mistaken as macrophages. Primary cultures of PCs isolated from brain microvessels, following isolation and cultivation of brain microvessels, may contain contaminating brain cell populations including PVMs. PCs were incorrectly identified as having PVM markers such as CD163, CD11b and vimentin due to some of these in vitro studies, but more recent studies have shown that PCs do not express these markers in vivo [17,20,21,22,23,24,25,26]. A further study called into question whether CD68-, CD163- and CD169-positive perivascular cells were indeed PCs in vivo, and using EM found that these cells were able to phagocytose carbon particles, while PCs did not, positively identifying them as PVMs instead [27]. PVMs have been ultimately distinguished from PCs and other cell types by their expression of markers such as CD68, CD163, and CD206 and their lack of platelet-derived growth factor receptor-beta (PDGFRB) or smooth muscle actin (SMA). Neuron-glial antigen 2 (NG2), often considered as a suitable PC marker for studies of PC biology in mice, is expressed in a small subpopulation of PVMs, making it an imprecise marker for differentiating these two cell types [24,25,26,28].

### 2.2. Distinguishing PCs from ECs

Difficulty in differentiating PCs from ECs is due in part to the misuse of markers, but also the complexity of their complementary roles within the BBB. Early nomenclature for PC and other cells associated with the vasculature, such as Rouget cells, adventitial cells, pericapillary cells, periendothelial cells, perivascular cells and mural cells, make it difficult to find out in “modern” terms which cell types were being observed and described in the early literature [12,29]. One early study claimed that both ECs and periendothelial cells expressed aminopeptidase N (CD13), while a different study found that BBB-specific expression of CD13 by PCs were regulated by the presence of ECs [30,31]. These results made it important for later studies to specifically look at expression of CD13 and PECAM1 (CD31) in PCs and ECs to determine the type of cell that expresses each surface marker in the brain. It is now recognized that CD13 is specifically associated with PC in the BBB, while CD31 is only found on ECs [12,29].

Due to the close proximity of PCs and ECs within the neurovascular niche, other markers, which appear to be of vascular origin or pattern, were often presumed to be specific EC markers, but later were found to be associated with PCs instead. One such example is γ-glutamyl transpeptidase (GGTP), which was historically used as a marker for ECs and brain capillaries, largely in view of its role in the formation of a selectively permeable endothelial membrane [32,33]. When a later study examined this marker in vitro using ECs, astrocytes and PCs, it has become clear that this molecule is produced exclusively by PCs, yet increased in the presence of ECs and astrocytes [34]. This finding was a strong indicator of the role that PCs play in the development of a selectively permeable endothelial barrier and how complex the interplay between PCs and ECs can be within the BBB [34].

PCs and ECs share complementary roles in the BBB and are involved in many of the same pathways including the Shh signaling pathway. The role of PCs in the Shh-mediated upregulation of TJPs is not very well understood, but recent studies have found that brain PCs are required to mediate the Shh-induced paracrine signaling on adjacent ECs [35]. Additionally, recent studies have shown that PCs, but not ECs as previously thought, are the sole cellular producer of netrin-1 at the BBB [8,10,36]. Besides its best-known roles in development, netrin-1 is now recognized as a necessary intermediate of the Shh pathway by promoting the GLI1 transcriptional factor to specifically upregulate TJP production [10]. While further investigation on the role of PCs in the Shh pathway is warranted, there have been many recent findings indicating the role of PCs in regulating endothelial tight junction formation.

### 2.3. Distinguishing PCs from Vascular Smooth Muscle Cells

Perhaps vascular smooth muscle cells (VSMCs) are the most difficult cell type to distinguish PCs from. One reason for this may be that PCs and VSMCs appear to have a shared, yet heterogeneous, developmental origin [37,38,39]. While the precise origin of PCs and VSMCs are yet unclear, it seems evident that PCs and VSMCs arise from common developmental origins limiting the usefulness of fate mapping and lineage tracing as a method of differentiation. Compounding the issue of separating PCs and VSMCs into distinct cellular populations is the morphological and regional heterogeneity in this family of cells itself. Over the last decade or so, the once bilateral view of PCs and VSMCs of the BBB has evolved to include circumferential VSMCs (c-VSMCs), stellate VSMCs (s-VSMCs), ensheathing PCs, mesh PCs, and thin-strand PCs, each with different morphologies and vascular associations [29,40]. Despite the difficulty; however, differentiating these populations and sub-populations of cells can be crucial for experimental design due to their possibly different functions.

The most widely accepted way to identify VSMC and PC subpopulations while limiting bias is through examination of the neurovasculature with which they are associated. In the brain, c-VSMCs are thought to be associated with arterioles, while s-VSMCs are thought to be associated with venules [29,40]. Ensheathing PCs are thought to be associated with precapillary arterioles, and postcapillary venules, while mesh PCs and thin-strand PCs are commonly associated with capillaries [29,40]. Neurovascular arterioles and venules are defined as 0th order vessels, while pre- and postcapillary vasculature is usually accepted to be comprised of first to fourth order vascular branches, everything beyond which is considered a capillary [29,40]. In mice, observation of the order, or degree of capillary branching can be obtained through multi-photon microscopy, but the elongated branching of human brain vasculature has limited the field’s ability to use the branching order as a conclusive method for determining vascular identity, and thus PC/VSMC identity, in human samples [40]. Some studies have attempted to use vascular diameter to differentiate vascular subtypes and thus separate PCs and VSMCs, but human vascular diameter can vary based upon age, sex, brain region, tissue processing technique and many other risk factors, making it an unreliable determinant of vascular identity [1,29]. The various subpopulations of PCs and VSMCs may have distinctive morphologies but choosing the correct markers to visualize them can be challenging [29,40].

Historically, PCs were considered to be PDGFRB-positive, while VSMCs were positive for both PDGFRB and SMA. However, recent studies have shown that there are subpopulations of PCs which are SMA+, limiting SMA’s use in differentiating these two cell types [29,40,41]. Other PC or VSMC markers, such as desmin, NG2, CD146, CD13, Tbx18, nestin and myosin heavy chain 11 (MYH11), have been used to study these cells, but each comes with its own set of drawbacks and reservations (Table 1). Desmin has been shown to be present only in subpopulations of both VSMCs and PCs in highly variable levels based on environmental conditions; NG2 is present in both VSMCs and PCs at variable levels but can also be found on a subset of macrophages and NG2-glia; and CD146, CD13, and Tbx18 have been often used as pan-PC markers but also mark VSMCs and are; therefore, poor markers for differentiating these cells [26,29,42,43,44,45,46]. Nestin is expressed by neuronal progenitor cells in the brain and only seen in subsets of neurovascular PCs. While MYH11 has been suggested as a VSMC specific marker, more research is needed to determine which subsets of VSMCs it may be present on [8,29,46,47].

Currently, there are no PC or VSMC specific markers which readily distinguish PCs and VSMCs, but a new in vivo labeling technique may be used to overcome this challenge. A recent paper showed that injection of a green fluorescent FluoroNissl dye, NeuroTrace 500/525, into a mouse’s brain stained only PCs without staining VSMCs [55]. While this may not be a practical option for imaging human brain, which is often formalin-fixed, the field continues to make advances towards specific differentiation of PC and VSMC.

### 2.4. Summary

Differentiating PCs from other vascular and perivascular cells can be complex, but it is made easier by examining cellular location and markers. A comprehensive list of the markers mentioned in this paper can be found in Table 1. Ultimately, a system of morphology, vascular location, and a series of markers is needed to differentiate physiological PC and VSMC subsets, but recent findings strongly indicate that PCs may undergo further sub-differentiation under pathological conditions, further complicating their identification and classification.

## 3. Pathological PC Subsets in the Human Brain

In addition to the complexities of identifying PC from other vascular and perivascular cell types, a pathological subset of neurovascular PCs has recently been identified [8,54]. Following nomenclature set by the cancer field, physiological capillary PCs were termed Type-1 Pericytes (PC1), while the pathological subset of these capillary PCs were termed Type-2 Pericytes (PC2) [54,58]. Both subsets can be found on brain capillary vessels of less than 10 µm in luminal diameter and of the same branching order, but whether these correspond to mesh PCs or thin-strand PCs is unclear [54].

### 3.1. Origin of PC2

In our recent study examining PCs in the brains of humans and nonhuman primates, it was found that uninfected infant rhesus macaques demonstrated few to no PC2 in cortical tissue, which contains almost exclusively capillary PC1 [54]. While PC populations shift during aging or simian immunodeficiency virus infection from PC1 to PC2-dominant populations, the total number of PCs remains largely unchanged except in the most advanced stages of disease where PC loss is observed [8,54]. One proposal which would explain this phenomenon is that PC1 are transitioning to a PC2 phenotype under pathological conditions in vivo. This phenomenon was demonstrated in vitro when SMA-negative primary human PCs became SMA-positive after treatment with TGF-β1 [53,59]. While it is tempting to speculate this change will increase the contractility of microvascular PCs and affect cerebral blood flow, it is yet unclear whether SMA expression leads to or reflects further permanent change in PC phenotype. Visualizing this transition in vivo in human tissue is not practically possible, but future studies in animal models may be able to confirm PC1-to-PC2 transition in vivo.

A PC1-to-PC2 transition is consistent with the environmental sensitivity attributed to PCs and their role in maintaining BBB homeostasis, but, thus far, appears to be limited in vivo to transitioning from one PC subset to another. Some studies have suggested that PCs may demonstrate multipotent or pluripotent capabilities acting as an adult stem cell in the CNS, but recent in vivo studies have had difficulty initiating these stem cell-like activities from PCs under standard physiological or pathological conditions [45,60]. While ability of PC1 to switch to PC2 in vivo has yet to be confirmed, it seems more likely than the alternative, which would be a replacement of PC1 by new PC2 originated from precursors.

### 3.2. Identifying PC2

Early studies of PCs were often limited by the lack of specific markers, due in part to the existence of PC subsets [17,34,58]. An early study looking at PCs in tumorigenesis introduced the idea of a pathological PC subset after noting different cellular markers for PCs associated with normal vasculature, and PCs associated with tumor vasculature [58]. In this study, the authors demonstrated that tumorigenic PCs, termed PC2, express SMA, but this is neither the first nor the last study to show the presence of SMA on capillary PCs [58].

Studies dating back to 1985 have shown the presence of SMA on a subset of capillary PCs, calling into question the traditional view that PCs were SMA-negative and VSMCs were SMA-positive [51,52]. One explanation for a series of conflicting reports in literature on the presence of SMA in capillary PCs would be the presence of a SMA+ pathogenic PC subtype [51,52]. More recent studies have elucidated more information on the differences between these two PC subtypes [46,54,61]. One study identified two distinct but unnamed PC subsets, one which is CD90-positive with limited expression of SMA, and one being CD90-negative with higher expression of SMA and PDGFRB [61]. Another study used mesenchymal angioblasts to induce the development of PC1, PC2, and VSMC from progenitors and found that PC1 express PDGFRB, but not SMA, while PC2 express SMA and PDGFRB. Both lacked a VSMC marker MYH11 [46]. This same study found that PC1 expressing VCAM1 and CD274 could distinguish PC1 from the DLK1-expressing PC2 [46]. The use of SMA and MYH11 in combination as distinguishing markers was recently confirmed in vivo when a PDGFRB+/SMA−/MYH11− phenotype was successfully used to identify PC1, PDGFRB+/SMA+/MYH11− for PC2, and PDGFRB+/SMA+/MYH11+ marked only VSMCs in the brains of rhesus macaques and humans [54]. While other markers, including nestin, are shown to be expressed in a subpopulation of PCs in the mouse brain [62], further research is needed to determine their expression profiles in PC1 versus PC2.

Not only do PC1 and PC2 have different markers, but data suggest that they likely also have different functions. Numerous studies have described morphological differences between PCs particularly in aging or diseased individuals [46,58,63]. When identifying the two distinct PC subsets, PC1 have the traditional thin bump on a log morphology with a small amount of extracellular matrix, while PC2 are hypertrophied with a greater amount of extracellular matrix and may contain dark granules [8,61,63,64]. These morphological differences may speak to differences in their function and help to elucidate their role in BBB homeostasis and disease.

### 3.3. Functional Differences between PC1 and PC2

Reaching a better understanding of the functional differences between PC1 and PC2 can be achieved by studying their presence in various states of BBB homeostasis and disease. PC1 fit the traditional description of BBB-supportive microvascular PCs, while being the most abundant subpopulation found in young healthy individuals [8,54]. They are rarely associated with areas of fibrinogen extravasation but are associated with an organized and regulated basement membrane and astrocytic endfoot arrangements [8,54]. PC2, on the contrary, are significantly increased in aging or diseased individuals and are commonly associated with vessels demonstrating irregular basement membrane and astrocytic endfoot arrangements, and fibrinogen extravasation [8,54].

One reason for this increase in BBB breakdown in PC2-associated vessels could be a PC2-mediated disruption of the Shh pathway. PC2 has been shown to have higher levels of netrin-1 expression than PC1, despite being associated with vessels containing lower levels of claudin-5 [8,54]. This increase in netrin-1 may be acting as a compensatory mechanism to make up for a down-stream deficiency in Shh signaling which is preventing the successful production of more TJPs. We note that additional TJP affecting pathways may be differentially regulated in PC1 and PC2, and that those also need to be explored. Understanding the unique roles that PC1 and PC2 are playing in maintaining BBB homeostasis could help to elucidate new ways to target this system and restore equilibrium, but the functional differences between PC1 and PC2 become even more pronounced in other models of disease.

**Figure 1:**
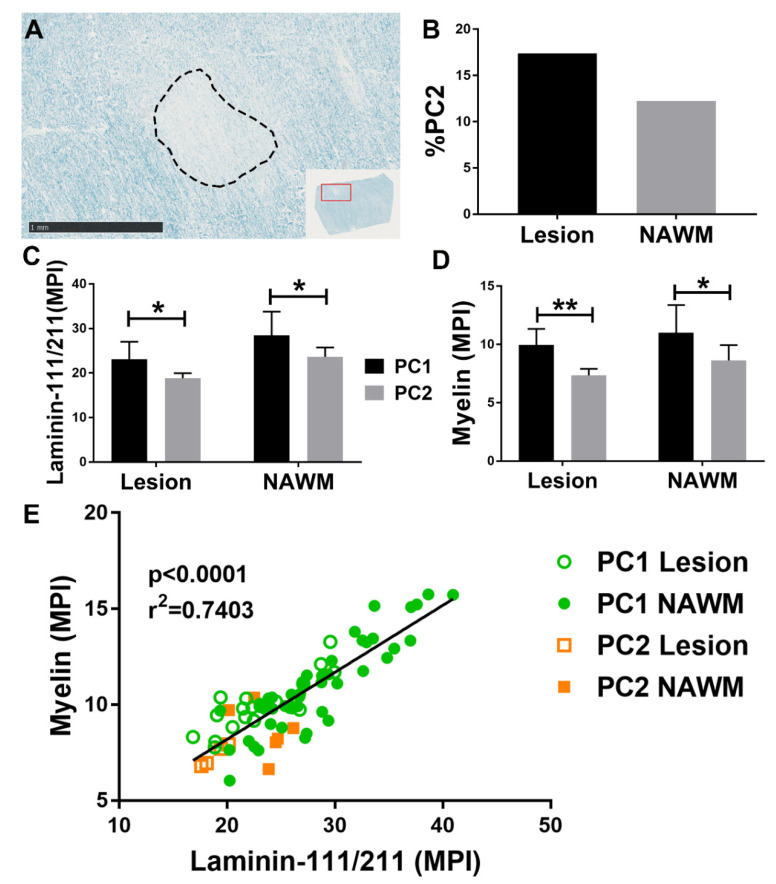
Luxol fast blue staining of a formalin-fixed paraffin-embedded corpus callosum tissue section, from a middle-aged female multiple sclerosis patient (obtained as de-identified from a commercial source, BioChain, Newark, California), shows a demyelinating lesion which is circled with a dashed line (**A**). A serial section was then stained with anti-PDGFRB-AF488, anti-SMA-AF647, anti-laminin-111/211-BV480 and anti-myelin-Cy3, and was examined under confocal microscopy. The number of PC1- and PC2-associated vessels were counted in five lesion and five NAWM frames at 40x and the %PC2 was calculated and indicated that there was a higher %PC2 in the lesion than NAWM (**B**). Regions of interest (ROIs) were established by outlining the PC1- and PC2-associated vessels from five 40x frames within the lesion and NAWM. The MPIs of laminin-111/211 and myelin staining were quantified in each ROI using NIH ImageJ to determine that PC2-associated vessels correlated with lower laminin-111/211 and myelin than PC1-associated vessels regardless of lesion association (**C**,**D**). A correlation between the laminin-111/211 and myelin MPIs between each ROI showed that there was a strong positive correlation between laminin-111/211 and myelin and that PC2-associated vessels fell only on the low end of that association (**E**). Graphs with statistical analysis were generated using GraphPad Prism: * = *p* < 0.05, ** = *p* < 0.01.

In vitro studies suggest that PC2 produces lower levels of laminin-111 (α1β1γ1) and laminin-211 (α2β1γ1), and loss of laminin-111 and laminin-211 has been shown to induce a PC2-like phenotype in PCs including hypertrophied morphology, increased SMA, BBB breakdown and reduced TJP protein production [3,4,46,65]. In addition, studies indicate that laminin-211 is necessary for oligodendrocyte precursor maturation and decreased levels is associated with myelin defects, which suggest a role for PC2 in demyelinating diseases like MS [13,14,46]. Herein we illustrate the utility of PC1/PC2 paradigm in studying PCs in the pathogenesis of MS (Figure 1). A demyelinating lesion from the corpus callosum of a middle-aged female MS patient (Figure 1A) had a higher %PC2 than the normal appearing white matter (NAWM) of the same patient (Figure 1B) [54]. The mean pixel intensity (MPI) of both laminin-111 and laminin-211 and myelin was lower in association with PC2-associated vessels than in PC1 vessels regardless of lesion association (Figure 1C,D). Interestingly, there was a strong positive correlation when comparing the MPI of myelin and laminin-111/211 associated with each vessel, but PC2-associated vessels were all clustered to the lower half of the plot showing a reduction in both myelin and laminin-111/211 (Figure 1E). N.B.: These N-of-1 data are used to demonstrate the utility of PC1/PC2 paradigm as a methodological platform for studying PCs and are not a report of scientific findings. Adequately powered studies are needed to evaluate the relationship between PC2, laminin-111/211, and demyelination.

### 3.4. Summary

Altered PC morphology and protein expression has been seen in association with neurocognitive decline and disease pathogenesis for decades sparking debate across the field about the phenotype and function of PCs which can readily and ultimately be explained by the presence of PC subsets, both physiological and pathological [8,46,51,52,54,58,61,63,64]. Recent studies find that changes in pathological PC subsets, like changes in %PC2, are present longitudinally in correspondence with disease progression [8,46,54,58]. This is in contrast to the loss of PCs, which occurs abruptly in very late stage disease, suggesting that PC2 may have a stronger influence on the development and pathogenesis of neurological diseases and disorders than PC loss.

## 4. Conclusions

PCs are an important and complex player in maintaining BBB microvasculature in both health and disease, but the identification of PCs within the neurovascular niche has had a convoluted history further complicated by an oversimplified view of pericytic hierarchy and architectural complexity. Many of the early discrepancies in PC literature may be explained by either misidentification of other cellular populations, or differences between PC subsets and their ability to transition from one subset to another under changing environmental conditions. With this novel insight comes new implications for the role of PCs in neurological diseases and disorders and a new framework within which we can study their impact on BBB homeostatic regulation and deterioration. Future research aiming to understand the role of PCs in brain physiology and pathology would benefit from novel techniques to investigate and differentiate them, as there is a substantial pool of novel information yet to be gained by investigating the role of PCs and PC subsets in human disease.

## Figures and Tables

**Table 1:** Summary of proteins discussed in this article and the selected cell types which express them.

Marker	Pericytes	Vascular Smooth Muscle Cells	Endothelial Cells	Perivascular Macrophages	Source
CD163	−	−	−	+	[24,25,27,48]
CD11b	−	−	−	+	[49]
Vimentin	−	−	+/−	+	[18,22,50]
NG2	+/−	−	−	+/−	[26,28]
CD206	−	−	−	+/−	[24,27]
CD68	−	−	−	+	[24,27]
CD13	+	+	−	−	[29,46]
CD31	−	−	+	−	[29,46]
GGTP	+	−	−	−	[34]
Netrin-1	+	+	−	−	[8]
PDGFRB	+	+	−	−	[29,40,41,46]
SMA	+/−	+	−	−	[21,46,51,52,53]
Desmin	+/−	+/−	−	−	[46]
CD146	+	+	+/−	−	[42,46]
Nestin	+/−	−	−	−	[46]
Tbx18	+	+	−	−	[45]
MYH11	−	+	−	−	[46,54]
Neuro Trace 500/525	+	−	−	−	[55]
DLK1	+/−	−	−	−	[46,49,56]
RGS5	+	+	−	−	[49,57]
KIR6.1	+	+	+	−	[49,57]
CD274	+/−	+	+	+	[46,49]

## Data Availability

The data presented in this study are available from the corresponding author upon reasonable request.
